# *In vivo* imaging of fluorescent single-walled carbon nanotubes within *C. elegans* nematodes in the near-infrared window

**DOI:** 10.1016/j.mtbio.2021.100175

**Published:** 2021-12-02

**Authors:** Adi Hendler-Neumark, Verena Wulf, Gili Bisker

**Affiliations:** a Department of Biomedical Engineering, Faculty of Engineering, Tel Aviv University, Tel Aviv, 6997801, Israel; b Center for Physics and Chemistry of Living Systems, Tel-Aviv University, Tel Aviv, 6997801, Israel; c Center for Nanoscience and Nanotechnology, Tel-Aviv University, Tel Aviv, 6997801, Israel; d Center for Light Matter Interaction, Tel-Aviv University, Tel Aviv, 6997801, Israel

**Keywords:** Caenorhabditis elegans, Autofluorescence, Single-walled carbon nanotubes, Near-infrared fluorescent nanoparticles, In vivo imaging

## Abstract

*Caenorhabditis elegans* (*C. elegans)* nematodes serve as a model organism for eukaryotes, especially due to their genetic similarity. Although they have many advantages like their small size and transparency, their autofluorescence in the entire visible wavelength range poses a challenge for imaging and tracking fluorescent proteins or dyes using standard fluorescence microscopy. Herein, near-infrared (NIR) fluorescent single-walled carbon nanotubes (SWCNTs) are utilized for *in vivo* imaging within the gastrointestinal track of *C. elegans*. The SWCNTs are biocompatible, and do not affect the worms’ viability nor their reproduction ability. The worms do not show any autofluorescence in the NIR range, thus enabling the spectral separation between the SWCNT NIR fluorescence and the strong autofluorescence of the worm gut granules. The worms are fed with ssDNA-SWCNT which are visualized mainly in the intestine lumen. The NIR fluorescence is used *in vivo* to track the contraction and relaxation in the area of the pharyngeal valve at the anterior of the terminal bulb. These biocompatible, non-photobleaching, NIR fluorescent nanoparticles can advance *in vivo* imaging and tracking within *C. elegans* and other small model organisms by overcoming the signal-to-noise challenge stemming from the wide-range visible autofluorescence.

## Graphical abstract

**Figure:**
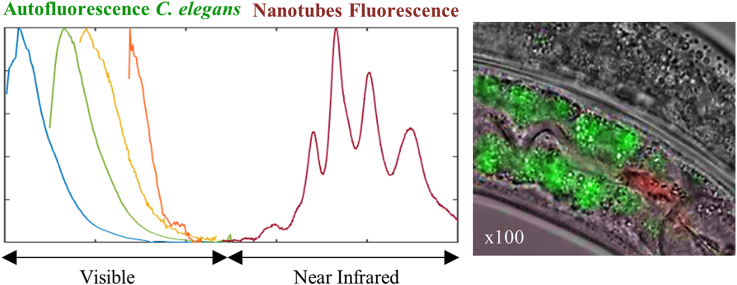

## 1 Introduction

Distinct organisms, such as nematodes, flies, or fish, are widely used as model systems in order to study human diseases, taking advantage of their small size and rapid life cycles combined with genomic similarities to higher eukaryotes. In contrast to single cells, these model systems allow for investigating the consequences of genetic, physiological, and developmental defects on all of the organs and tissues of an organism [1]. *C. elegans* are free-living nematodes, which serve as an excellent experimental model organism for numerous applications [2,3]. Around 60–80% of the human protein coding genes have predicted orthologues in *C. elegans* [2]*,* 40% of which are known to be associated with human diseases [1,4]. Due to their high genome similarity and the fact that generating mutant worms or recombinant inbred lines is quite easy, they can serve as a model for human diseases [5,6]. For example, *C. elegans* have been successfully used as a model for research on Huntington disease, which is a progressive neurodegenerative disease caused by polyglutamine (polyQ) repeat expansion in the Huntington protein [7]. A model of polyQ neurotoxicity in *C. elegans* was generated by expressing N-terminal fragment from the human Huntington that cause polyQ-dependent degeneration of neurons [8].

Owing to their small size (0.25–1 ​mm) and optical transparency, processes inside the worms, or their developmental stages, can be examined under a simple dissection microscope, or a confocal microscope which allows even finer resolution [1]. In order to image processes inside the worms, certain proteins, cellular compartments, or tissues, are labeled with fluorescent markers. Moreover, autofluorescence of the worm itself can serve as a reporter [[9], [10], [11]] to indicate health, aging, and developmental stages of the organism or specific organs [[12], [13], [14], [15]]. Strong autofluorescence in *C. elegans* can be detected throughout the visible spectral range, due to two major contributors located in the uterus and the intestine. In the uterus, yolk proteins accumulation in unfertilized oocytes are the source of the autofluorescence, which is further enhanced in a specific age of the animal when the uterus if filled with eggs [12]. However, most of the autofluorescence in *C. elegans*, as in mammalian cells, is found in the granules of lysosomes, which in case of the worms, are confined to their intestinal system. The intestine fluorescent compound is Lipofuscin, which is located in the gut granules, the secondary lysosome. The amount of Lipofuscin increases with the age of the worm, thus providing a versatile indicator for aging [13,14,[16], [17], [18], [19]].

**Scheme 1:**
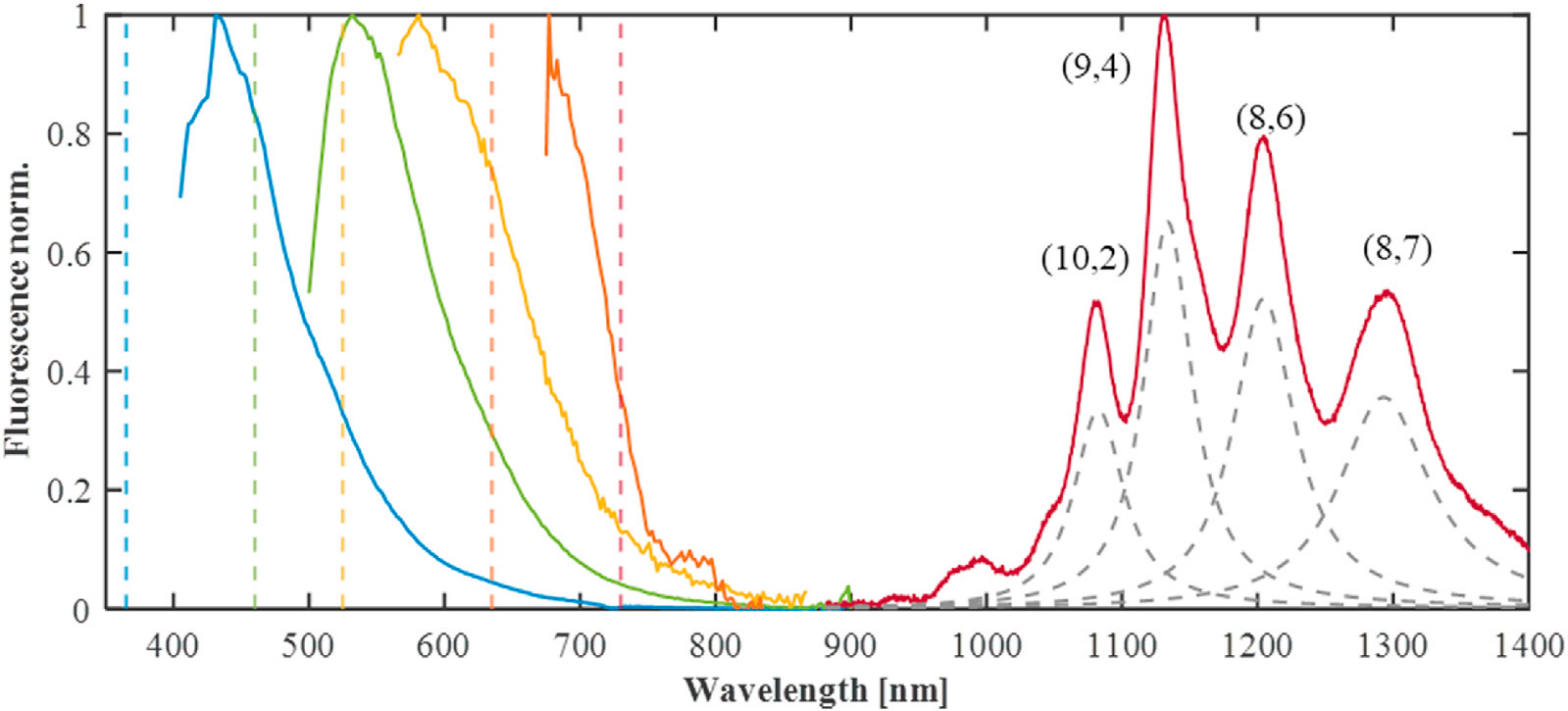
**Visible autofluorescence of *C. elegans* and NIR fluorescence of SWCNTs.** Visible autofluorescence (solid blue, green, yellow, and orange curves, 405–900 ​nm) of the worms was measured with several excitation wavelengths (dashed lines). The worms show autofluorescence in the entire visible range up to 900 ​nm. The SWCNT fluorescence (solid red curve, >900 ​nm) inside the worms does not overlap with the *C. elegans* autofluorescence. Line colors: blue (λ_ex_ ​= ​365 ​nm; λ_em_ ​= ​405–900 ​nm), green (λ_ex_ ​= ​460 ​nm; λ_em_ ​= ​500–900 ​nm), yellow (λ_ex_ ​= ​525 ​nm; λ_em_ ​= ​565–900 ​nm), orange (λ_ex_ ​= ​635 ​nm; λ_em_ ​= ​675–900 ​nm) and red (NIR-λ_ex_ ​= ​730 ​nm; λ_em_ ​= ​900–1420 ​nm). The excitation in the visible range was done in a fluorescence plate reader (Fusion Optics Reader Platform SPARK), and the excitation of the NIR fluorescence was done with a 730 ​nm CW laser. The autofluorescence and fluorescence spectra were normalized by their peak values to highlight the corresponding wavelength ranges. The grey dashed lines show the different SWCNT chiralities contributing to the spectrum as a guide to the eye.

In addition to studies based on the autofluorescence of the intestines or the uterus of the worms, fluorescent markers are utilized to report defects in function, gene-expression, development, or protein interactions *in vivo* [9,20]. One of the most common reporter proteins introduced into worms is the green fluorescent protein (GFP) [21]. A library of transgenic *C. elegans* that has a promoter fusion to GFP in thousands of genes is available nowadays, due to a high throughput cloning and expression project. Such transgenic worms have made a big contribution to visualizing processes such as protein localization, cellular identification, anatomy, and visualizing physiological processes [9,22]. Worm cell compartments can be visualized with a variety of fluorescent dyes like 4′,6′-diamidino-2-phenylindole hydrochloride (DAPI) for DNA [23], lipophilic dyes for exposed neurons [24,25], and fluorescein-conjugated phalloidin for actin filaments [26]. Besides protein labeling in *C. elegans*, fluorescent bacteria also contribute to research on organism toxicity and host-pathogen interactions, like bacterial virulence. These bacteria usually express a fluorescent protein and are introduced into the worms via food uptake to identify the defense response pathway and the interaction of the offensive and defensive factors involved [[27], [28], [29], [30], [31]]. For example, in worms fed with mCherry-labeled *Salmonella*, the bacteria generated aggregates or biofilms in the gut as a survival strategy [32]. The use of fluorescent proteins or bacteria can be challenging due to the fact that in some techniques, like fluorescence correlation spectroscopy, appropriate expression levels of proteins are critical for the suitability of the method, whereas excess expression levels may contribute to artifacts [33]. GFP and mCherry, like most common fluorescent dyes, show fluorescence emission in the visible wavelength range, overlapping with the autofluorescence of the worms. As the autofluorescence emission occurs in the entire visible range (Scheme 1), fluorescent proteins can unambiguously label only proteins or compartments that are spatially separated from the intestines and the uterus. One approach to overcome this challenge is feeding RNA interference (RNAi) to the worms, which can lower the autofluorescence of the intestine, but at the same time, can result in alterations in gene functions [16,20]. Another option is to address the fluorescent crosstalk technically using a triple band filter in the microscope, which spectrally separates between the emission of the fluorescent protein and the autofluorescence. However, such filters also block some of the fluorescence emission of the dye [34]. One emerging option to overcome fluorescence masking, is to utilize fluorescent markers whose emission are spectrally separated from the autofluorescence of the worms.

Semiconducting single-walled carbon nanotubes (SWCNTs) are fluorescent nanostructures emitting in the near infrared (NIR) spectral region, mainly between 900 and 1400 ​nm [35] (Scheme 1). SWCNTs can be thought of as graphene sheets rolled up into cylindrical structures. Differences in their ‘roll-up’ vector give rise to different chiralities of SWCNTs with different diameter and optical transitions [36]. Each of these chiralities has a resonant fluorescence excitation (E_22_-transition) and emission (E_11_-transition) wavelength, which can function as independent and specific fluorescent probe or sensor given proper functionalization [[37], [38], [39], [40], [41]]. The SWCNT benefit from large stokes-shifts with excitation wavelength in the visible wavelength range, and emission wavelength in the NIR biological transparency window (>900 ​nm) [[42], [43], [44], [45], [46], [47], [48], [49]].

Despite being highly hydrophobic nanostructures, SWCNTs can be easily suspended in aqueous solution using noncovalent functionalization by polymers, DNA, RNA, dendrons, proteins, peptides, or specific recognition elements like antibodies or aptamers [[50], [51], [52], [53], [54], [55], [56], [57], [58], [59], [60], [61], [62], [63], [64], [65], [66], [67]]. Only functionalized, suspended SWCNTs reveal a fluorescence signal. Several studies demonstrated the feasibility of SWCNTs as fluorescence sensors or markers *in vivo* [[46], [47], [48],^51^,^59^,[68], [69], [70], [71], [72], [73], [74], [75], [76]]. For example, single nanoparticle tracking of SWCNTs in the extracellular space of live brains could locally resolve its dimensions and viscosity [77,78]. Moreover, a nitric oxide sensor for epidermal tissue inflammation was demonstrated in mice [42], and experiments with larger animal models are emerging [69,79].

The NIR fluorescence emission of SWCNTs for imaging in *C. elegans* can be spectrally separated from the autofluorescence of the worms, hence, its application is not limited to certain compartments. Further, in contrast to organic fluorescent dyes, SWCNTs show no photobleaching nor blinking [[80], [81], [82]], and thus can be used for the imaging of dynamic processes over long timespans.

In this work, we present the feasibility of utilizing SWCNTs as fluorescent probes in *C. elegans*. We show that the fluorescence emission in the NIR does not overlap with the autofluorescence of the organelles of the worms, and thus, can be used to image processes in the entire organism without genetic modifications. Further, the presence of selective markers or protein tags in the final strain can lead to misleading phenotype interpretation due to the neighboring gene effect [83,84]. The model system of DNA suspended SWCNTs used in this study, does not show toxicity or any adverse effects. The SWCNT concentration can be easily tuned to fit the optimal signal levels and the limit of detection required for a specific experimental method. SWCNTs taken up by the worms are imaged inside the animal and their movement can be monitored over time, owing to their photostable fluorescence emission, opening up the possibility of sensing analytes *in vivo* [85,86]. SWCNT samples contains different chiralities, each of them with a specific resonance excitation and emission wavelength (Scheme 1). Different functionalization of the various chiralities, can thus create a pool of NIR-fluorescence sensors in different wavelengths. Our results pave the way to new applications of fluorescent probes and nanosensors for bio-imaging in the *C. elegans* research community. Moreover, due to their fluorescence in the biological transparency window, these optical nanoprobes can be transferred to other *in vivo* systems.

## 2 Results

### 2.1 No autofluorescence of *C. elegans* in the near-infrared

The applicability of SWCNTs as fluorescence probes in *C. elegans* relies on the premise that the autofluorescence of the worms is limited to the visible wavelength range (Scheme 1). SWCNTs reveal fluorescence emission in the NIR range, corresponding to their E_11_-transitions, >900 ​nm, while the excitation of the E_22_-transitions can range from the visible wavelengths up to 900 ​nm [36,87] (Scheme 1).

**Fig. 1:**
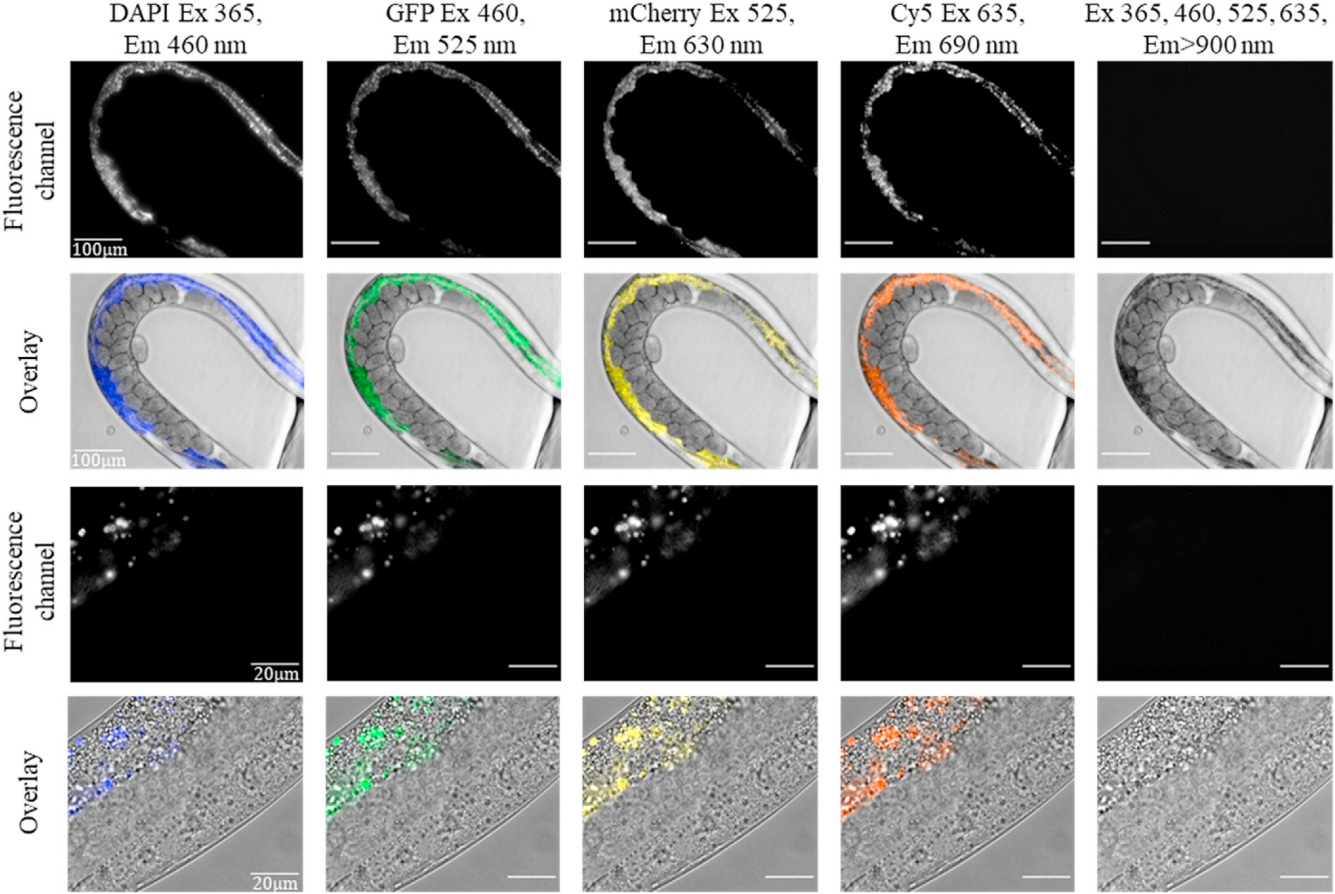
**Autofluorescence of *C. elegans* in the visible and NIR spectral range.** Visible autofluorescence (400–800 ​nm) of the worms was imaged with an EMCCD-camera under LED illumination (4 leftmost columns). The fluorescence channels imaged are named after the common dyes they are applied to, and cover the visible emission wavelength range: DAPI (blue, λ_ex_ ​= ​365 ​nm; λ_em_ ​= ​435–485 ​nm), GFP (green, λ_ex_ ​= ​460 ​nm; λ_em_ ​= ​500–550), mCherry (yellow, λ_ex_ ​= ​525 ​nm; λ_em_ ​= ​590–670 ​nm) and Cy5 (orange, λ_ex_ ​= ​635 ​nm; λ_em_ ​= ​665–715 ​nm). The right column shows images taken with an InGaAs camera, detecting fluorescence in the NIR spectral region (λ_em_ ​> ​900 ​nm) under LED illumination with the four wavelengths used for the visible range (λ_ex_ ​= ​365, 460, 525, 635 ​nm), demonstrating the lack of autofluorescence in the NIR. The excitation power and exposure time for the respective images are summed up for comparison in Table S1. Rows 1 and 3 show the fluorescence emission images, rows 2 and 4 show their overlay with the brightfield images. The scale bar in the top two rows is 100 ​μm and in the bottom two rows 20 ​μm.

We monitored the autofluorescence of the worms in the visible and the NIR wavelength range using an LED illumination system covering the visible range (Fig. 1). The autofluorescence of the worms was captured with two different cameras, an EMCCD for the visible wavelength range (400–800 ​nm) and an InGaAs camera for the NIR range (900–1700 ​nm). The two cameras have similar quantum efficiency (>80%) in their respective wavelength ranges and can therefore be used to compare the detected fluorescence signal.

The autofluorescence emission of the worms in the visible wavelength range was separated into 4 spectral regions with fluorescence excitation emission filter cubes corresponding to common fluorescent dyes: DAPI (λ_ex_ ​= ​365 ​nm; λ_em_ ​= ​435–485 ​nm), GFP (λ_ex_ ​= ​460 ​nm; λ_em_ ​= ​500–550 ​nm), mCherry (λ_ex_ ​= ​525 ​nm; λ_em_ ​= ​590–670 ​nm), and Cy5 (λ_ex_ ​= ​635 ​nm; λ_em_ ​= ​665–715 ​nm). The worms show strong autofluorescence emission in all four wavelength ranges (Fig. 1) emphasizing the challenge of overlapping emission signals of the autofluorescence and common fluorescent dyes. The fluorescence in the NIR channel in our setup is transmitted through a 900 ​nm long-pass dichroic mirror and a 900 ​nm long-pass emission filter separating the visible excitation light from the NIR emission and then captured by the InGaAs camera. Under simultaneous excitation with all four LEDs (λ_ex_ ​= ​365, 460, 525, 635 ​nm) used to excite the autofluorescence in the visible range, no fluorescence signal was observed in the NIR (λ_em_ ​= ​900–1700 ​nm) (Fig. 1). Additionally, we imaged the worms under excitation with a supercontinuum laser covering the entire visible wavelength range between 400 and 850 ​nm, but again no fluorescence emission was detectable in the NIR-camera (Figure S1). As the imaging of the SWCNTs is done under illumination with a CW-laser with an excitation wavelength of λ_ex_ ​= ​730 ​nm and a higher output power than the LEDs or the supercontinuum laser (see Table S1), we also confirmed that no autofluorescence could be detected with the CW-laser with the same exposure times (t_ex_ ​= ​1–2 ​s) used for imaging the SWCNTs (Figure S2). The excitation power values of all light sources at their respective wavelengths are summarized in Table S1. These results indicate that the fluorescence emission in the NIR region of the SWCNTs can indeed circumvent interference with the autofluorescence of the worms.

### 2.2 DNA-SWCNT biocompatibility in *C. elegans*

The toxicity of functionalized nanostructures can stem from both the material of the nanoparticle and its surface functionalization [88,89]. SWCNTs have been widely used for imaging and sensing applications and were shown to have high biocompatibility [42,48,72]. For our study, we chose a single-stranded DNA functionalization, (GT)_15_-SWCNTs, as a model system owing to the ease of sample preparation and biocompatibility [72,90].

We incubated the worms with (GT)_15_-SWCNT, with concentrations ranging from 0.1 to 300 ​mg ​L^−1^, or with 0.1 ​M NaCl as control, for 24 ​h. Even with the highest concentration used, the vast majority of the worms remained viable, having similar mobility as the control sample (Supplementary Movie S1 and S2).

Supplementary data related to this article can be found online at https://doi.org/10.1016/j.mtbio.2021.100175

The following are the Supplementary data related to this article:

Multimedia component 1 Multimedia component 1

Multimedia component 2 Multimedia component 2

**Fig. 2:**
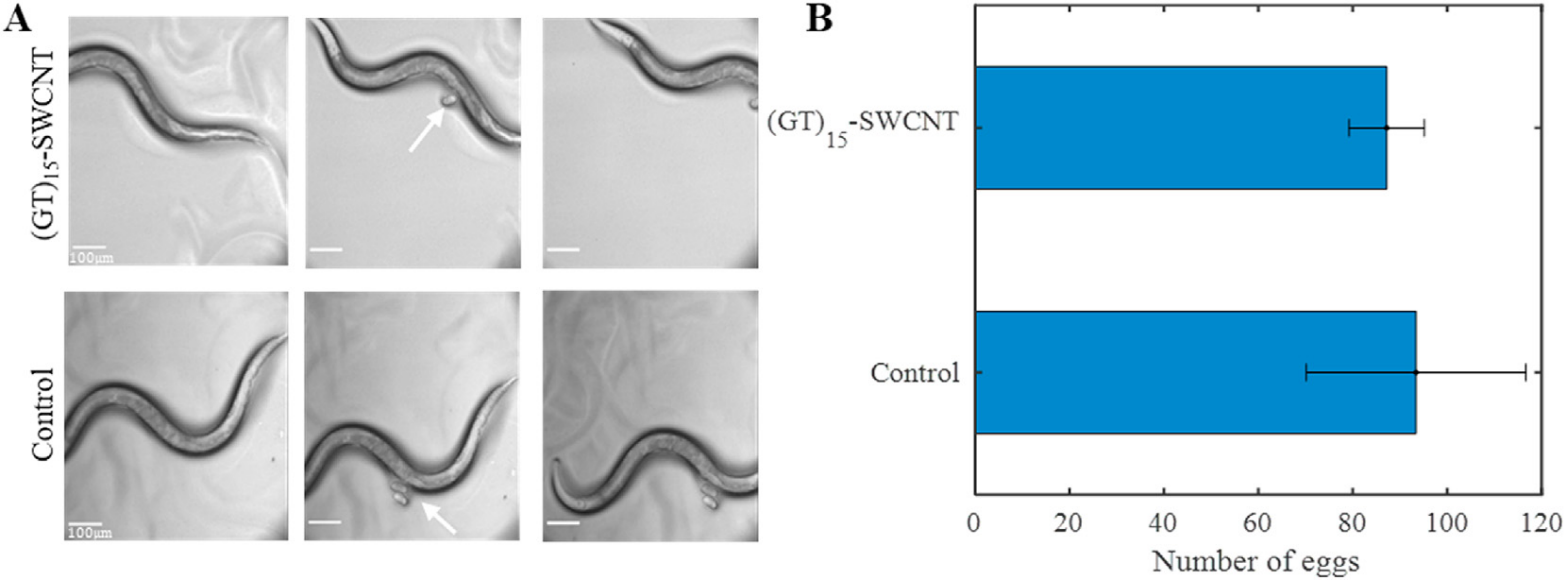
**SWCNTs do not influence mobility or the ability of the worms to lay eggs. A**) Brightfield images adapted from time-lapse movies of the worms 4 ​h after the exposure to (GT)_15_-SWCNT (Supplementary Movie S3) or 0.1 ​M NaCl (Supplementary Movie S4) as control. White arrows point to eggs laid by the worms during the experiment. The scale bar is 100 ​μm. **B**) The number of eggs counted on the plates after 4 ​h of exposure to (GT)_15_-SWCNT or 0.1 ​M NaCl as control. The results show the average over four independent plates.

To determine the working concentration of the SWCNTs for imaging, we incubated the worms in 0.5, 5, 25, or 100 ​mg ​L^−1^ ​(GT)_15_-SWCNT for 4 ​h. The optimal signal-to-noise ratio in the NIR images was obtained for 0.5 ​mg ​L^−1^ ​(GT)_15_-SWCNT. Lower concentrations resulted in a weak signal, whereas higher concentrations resulted in high background fluorescence from SWCNT binding to the surface of the worms. After choosing the working SWCNT concentration (0.5 ​mg ​L^−1^) for imaging, the (GT)_15_-SWCNT biocompatibility was further confirmed by testing the worms mobility and reproduction ability, i.e. the ability to lay eggs, following exposure to the nanotubes [[91], [92], [93]]. 10 adult worms were transferred to fresh culture plates which were spread either with 0.5 ​mg ​L^−1^ ​(GT)_15_-SWCNT or 0.1 ​M NaCl as a control. After 4 and 24 ​h, the worms were assessed by observing their motion under the microscope (Supplementary Movie S3-6), showing similar mobility of the worms exposed to SWCNTs compared to the control group (Fig. 2A). Further, the number of eggs laid during the first 4 ​h was 94 ​± ​11 for the worms exposed to SWCNTs, and 93.4 ​± ​23.2 eggs for the control group (Fig. 2B), confirming there is no effect of the SWCNTs neither on their reproductive abilities nor on their mobility. Longer-term toxicity was tested by incubating the worms with 5 ​mg ​L^−1^ ​(GT)_15_-SWCNT, or 0.1 ​M NaCl as control, for 4 days. Following the incubation, the worms showed similar viability and mobility to the control (Supplementary Movie S7-8), and they continued to lay eggs as expected in both groups. These results demonstrate the biocompatibility of SWCNTs in the organism [94].

Supplementary data related to this article can be found online at https://doi.org/10.1016/j.mtbio.2021.100175

The following are the Supplementary data related to this article:

Multimedia component 3 Multimedia component 3

Multimedia component 4 Multimedia component 4

Multimedia component 5 Multimedia component 5

Multimedia component 6 Multimedia component 6

Multimedia component 7 Multimedia component 7

Multimedia component 8 Multimedia component 8

### 2.3 SWCNT fluorescence imaging alongside the autofluorescence of the worms

**Fig. 3:**
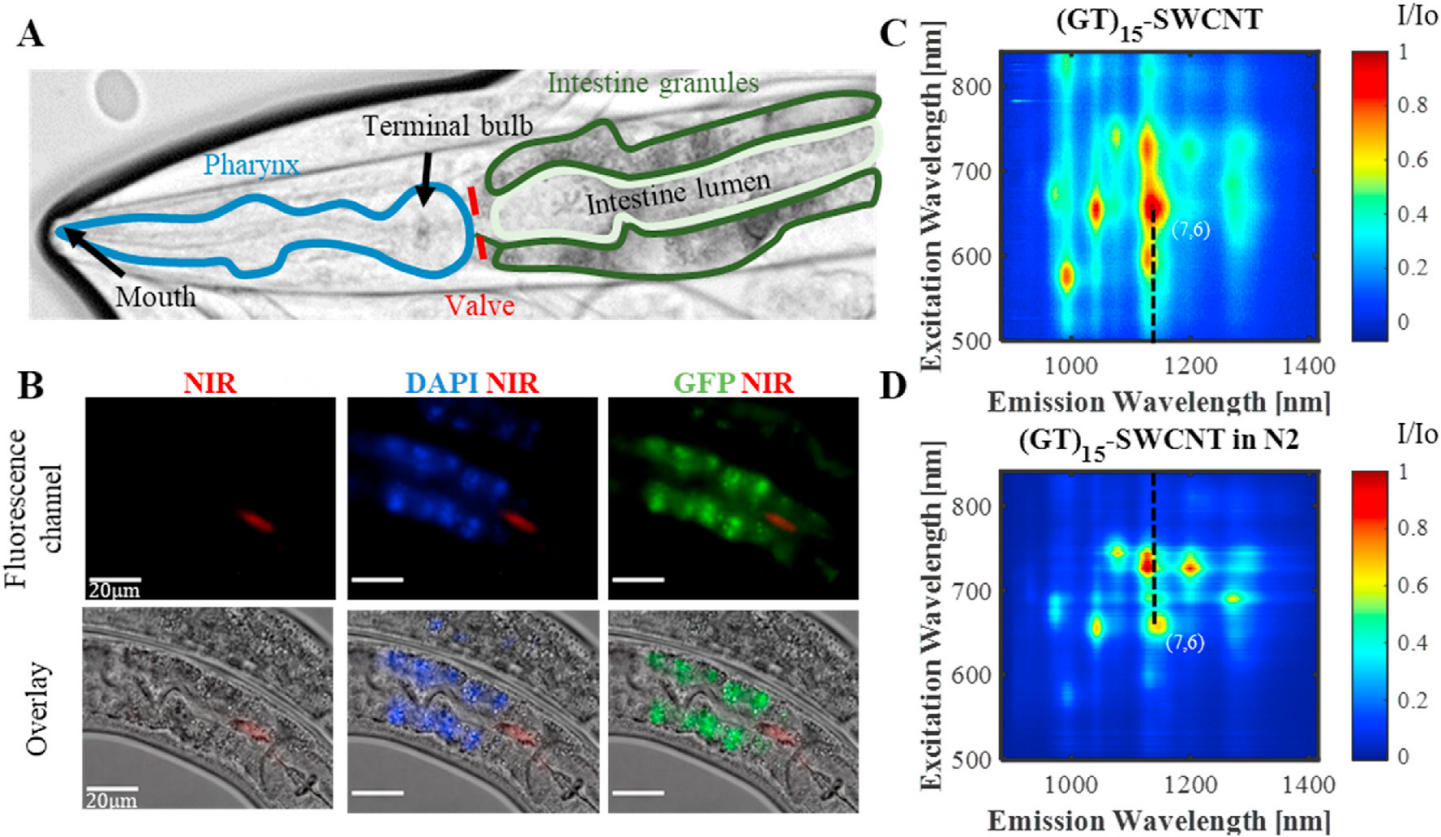
**Imaging SWCNT fluorescence within the worms. A**) *C. elegans* anatomy: the pharynx (blue), the intestine lumen (light green) and granules (dark green). The terminal bulb and the intestine lumen are separated by the pharyngeal valve (red). **B**) Fluorescence images of the SWCNTs in the NIR (red), SWCNTs with autofluorescence in the DAPI channel (red and blue), and SWCNTs with autofluorescence in the GFP channel (red and green) on the top row. The bottom row is the overlaid images of the brightfield and the fluorescence. The scale bar is 20 ​μm. The SWCNT-fluorescence is excited with the CW laser at λ_ex_ ​= ​730 ​nm while the autofluorescence was excited with the LED illumination system. **C**) Normalized fluorescence excitation emission map of 0.5 ​mg ​L^−1^ ​(GT)_15_-SWCNT and **D)** (GT)_15_-SWCNT within the N2 worms. The black dashed line shows the wavelength shift of the (7,6) chirality.

Having established that the autofluorescence of the worms does not spectrally overlap with the NIR fluorescence emission of the SWCNTs on the one hand, and proved the biocompatibility of (GT)_15_-SWCNT on the other hand, we imaged the SWCNT *in vivo* after they were taken up via food-intake by the worms. *C. elegans* worms take up food through their mouth by pumping the surrounding bacteria-containing liquid into the intestines via a tube-like muscle (pharynx) [95]. From the end of the pharynx (terminal bulb), the food reaches the lumen of the anterior intestines via a valve (Fig. 3A).

We incubated the worms with 0.5 ​mg ​L^−1^ ​(GT)_15_-SWCNT, or 0.1 ​M NaCl as control, for 4 ​h. Subsequently, the worms were rinsed to remove SWCNTs from the medium and imaged under the fluorescence microscope. We imaged the visible autofluorescence emission at the wavelengths λ_em_ ​= ​435–485 ​nm (DAPI) and λ_em_ ​= ​500–550 ​nm (GFP), which indicate the location of the intestine granule organelles within the intestinal cells. The NIR-fluorescence of the SWCNTs taken up by the worms was imaged in a wavelength range of λ_em_ ​= ​900–1700 ​nm. The overlaid brightfield and fluorescence images show that the SWCNTs pass the pharynx [96] and reach the lumen of the intestines, which is surrounded by the intestine granules, just after the terminal bulb [95,97] (Fig. 3B and S3). Although the SWCNTs are located in a compartment of the worm with strong autofluorescence, we can clearly identify their position due to their spectrally distinct fluorescence emission.

In order to characterize the changes in the fluorescence emission of the SWCNT after internalization into the worms, we measured the SWCNT fluorescence emission spectra between 900 and 1400 ​nm, for excitation wavelengths between 500 and 850 ​nm before and after incubation with *C. elegans* (Fig. 3C and D). These measurements were performed in a setup of a fluorescence microscope coupled to a spectrograph, and thus, represent the average SWCNT-spectra within the worms in the sample. After the incubation of the worms with the SWCNTs and their uptake into the worms, bright fluorescence of the different chiralities can still be observed, indicating that the SWCNTs do not suffer from significant aggregation within the worms (Fig. 3D and S4). Additionally, after incubation of the worms with the SWCNTs, we observe a redshift of the emission peak of the (7,6) chirality, which can be attributed to surface adsorption of proteins and other biomolecules present in the worm gut, in agreement with previous observations of solvatochromic shifts resulting from nonselective adsorption onto the SWCNTs [54,98]. Further, since we mainly observe the nanotubes inside the worm gut, where the pH is about 5 [23], the pH sensitivity of the SWCNTs fluorescence can also contribute to the observed shift [99].

### 2.4 Imaging real-time SWCNT mobility and dynamics within *C. elegans*

**Fig. 4:**
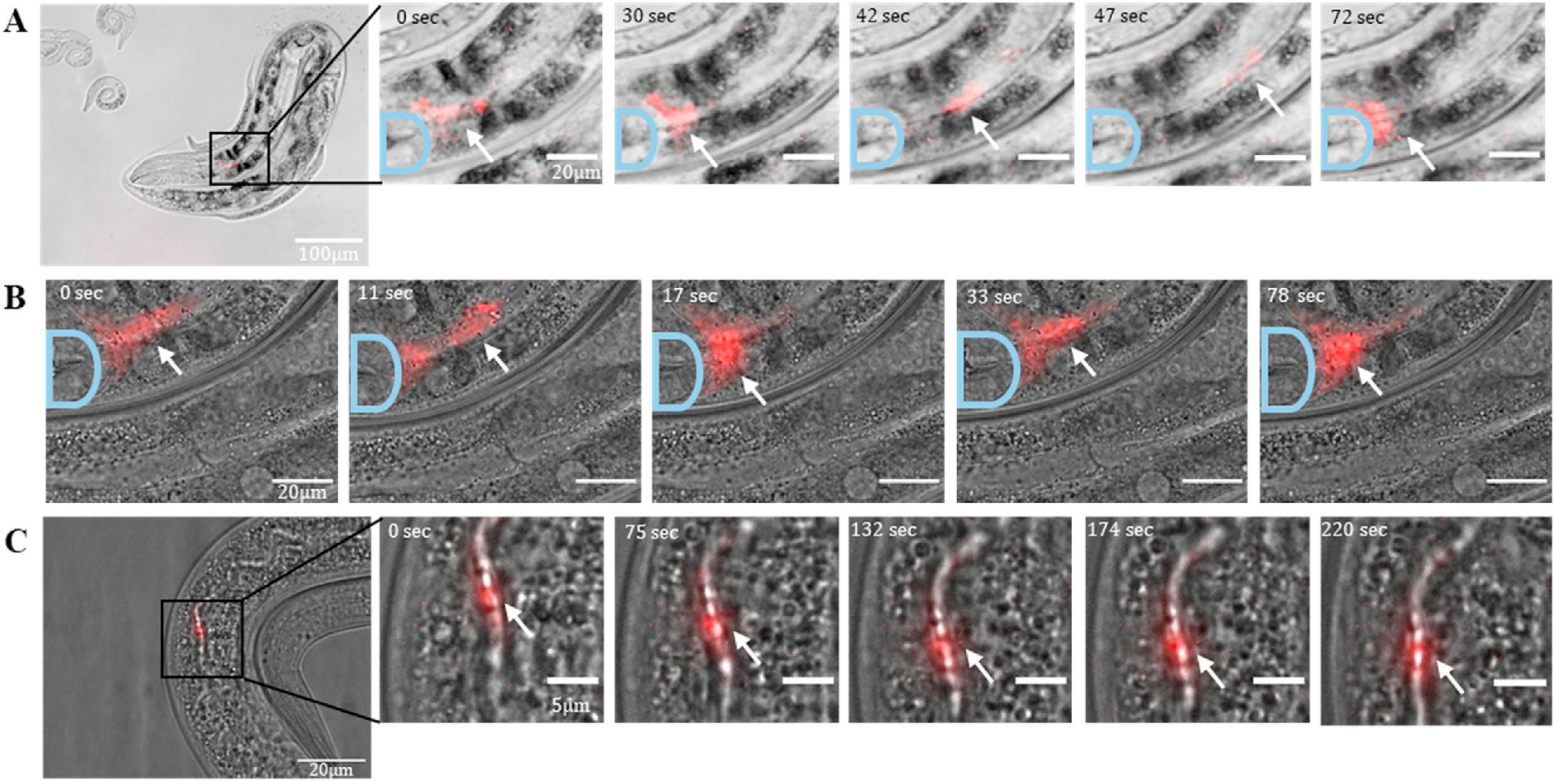
**SWCNTs image dynamics in the worms.** Brightfield images overlaid with NIR fluorescence images adapted from movies of the worms after 4 ​h of exposure to (GT)_15_-SWCNT. The red color is the (GT)_15_-SWCNTs fluorescence excited with the 730 ​nm CW laser (181–189 ​mW), and captured by a NIR camera (1000 msec exposure time). The white arrows show the SWCNT distribution and accumulation. The light blue line marks the terminal bulb. **A)** Images adapted from a movie taken with a 20× objective and the scale bar is 100 ​μm in the first image. The other images in the row show the ROI marked in the first image with a scale bar of 20 μm. (Supplementary Movie S9), **B)** and **C)** Images adapted from movies taken with a 100× objective and the scale bar is 20 ​μm for **B** and the first image in **C.** The other images in **C** show the ROI marked in the first image with a scale bar of 5 μm. (Supplementary Movie S10 and S11).

One of the main advantages of using single-walled carbon nanotubes as fluorescent probes instead of organic fluorescent molecules is that SWCNTs do not suffer from photobleaching [80]. Thus, they enable imaging of dynamic processes inside an organism over a long period of time [42,100]. Firstly, in order to rule out any heating effects, we assured that there was no temperature change of our samples within our experimental time-scale of several minutes. Further, even following an hour of continuous CW laser irradiation, we did not observe any significant temperature change beyond 2 ​°C, which is well within the optimal temperature range for *C. elegans* [23]. Accordingly, we could follow the fate of the (GT)_15_-SWCNT after their uptake by the worms, and benefit from the lack of autofluorescence background. Fig. 4 and S5 show snapshots from movies (Supplementary Movies S9-11) of the SWCNTs inside the worms after 4 ​h incubation. The nanotubes were digested through the pharynx and the pharyngeal valve into the lumen of the intestine as they can be clearly localized after the pharyngeal grinder, at the anterior of the terminal bulb or in the intestine. Over time, we can see periodical distribution and accumulation of the SWCNTs at the anterior of the terminal bulb. This process shows the pumping motion, i.e. muscle contraction of the pharynx, which lead to high pressure in the terminal bulb, forcing food through the pharyngeal-intestinal valve into the intestine (Fig. 4A and B) [96]. Moreover, the movement of the SWCNTs in the intestine shows the digestion process in the worms (Fig. 4C). The dynamic movement of the particles can be followed by analyzing the intensity of the signal to track the digestion process from the entrance to the intestine gut until contraction of the muscles inside the intestinal organs (Figure S5). The videos were taken in the NIR fluorescence channel with the InGaAs camera, where each frame was analyzed in terms of the number of pixels whose intensity exceeds a threshold (Figure S5A-F), or the coordinate of their center of mass (Figure S5G-I), in order to monitor the nanotubes movement. We find the periodicity of the nanotubes’ distribution in the pharyngeal-intestinal valve to be within the range of 10–20 ​s. These results demonstrate that SWCNTs can not only be used as fluorescence markers for tissue, compartments, or organelles, but also to image dynamic processes, like the muscular contraction that are taking place inside the worm, without being limited by photobleaching.

Supplementary data related to this article can be found online at https://doi.org/10.1016/j.mtbio.2021.100175

The following are the Supplementary data related to this article:

Multimedia component 9 Multimedia component 9

Multimedia component 10 Multimedia component 10

Multimedia component 11 Multimedia component 11

## 3 Discussion and summary

*C. elegans* are commonly used as a model organism to study environmental toxicity, human diseases, host-parasite interactions, and evolution [4,8,31,32]. This organism displays a high degree of conservation with humans at the molecular and cellular level and is easy to genetically manipulate for research [1,2,23].

The worms reveal strong autofluorescence over the entire visible wavelength range in their uterus and intestine, which take up the majority of the worm volume and thus interfere spatially and spectrally with most fluorescent dyes. In contrast, no autofluorescence is observed in the NIR wavelength range (>900 ​nm) from any of the organelles. The (GT)_15_-SWCNTs do not show any signs of short or long-term toxicity, as the worms’ mobility, viability, and reproductive ability are similar to a control group. Even incubation of 24 ​h in high concentrations of nanotubes (up to 300 ​mg ​L^−1^) did not show any change in viability compared to the control. Owing to the biocompatibility of the SWCNTs, we imaged the nanotubes inside the worms after they were taken up via food-intake. The SWCNTs were processed to the lumen of the intestines where they can be imaged in the NIR alongside the strong visible autofluorescence of the gut granules. The fluorescence intensity of the SWCNT within the worms can be controlled by tuning the SWCNTs concentration, without worrying about toxicity, in order to match the required intensity levels for a specific application. While SWCNT imaging within the worms could be done by LED excitation, we chose to excite with a laser to benefit from higher excitation intensity, better signal-to-noise, and shorter exposure times. The laser excitation did not show any phototoxic effect, and no heating effect was observed. Following their uptake, the SWCNT NIR fluorescence spectra could be resolved, assuring no massive aggregating occurred. The bright fluorescence signal of the SWCNT within the worms open the possibility of *in vivo* biosensing. Moreover, owing to the lack of photobleaching, and their spectral isolation in the NIR wavelength range, SWCNTs can be utilized to image dynamic processes inside the worm, like the pumping movement of the pharynx, without interfering autofluorescence.

Finally, fluorescent SWCNTs imaged inside an optically transparent model system like *C. elegans* can be easily transferred to higher order eukaryotes, i.e., to less transparent organisms, due to the fluorescence emission in the NIR transparency window of biological tissue. SWCNTs can be used for detecting several analytes or proteins simultaneously due to the fact that different chiralities have different excitation and emission wavelength, which do not overlap with the autofluorescence emission. Our results open numerous possibilities for imaging the gastrointestinal motility in *C. elegans* nematode worms, and other model organisms using NIR fluorescence microscopy.

## 4 Material and methods

### 4.1 *C. elegans* growth and maintenance

*C. elegans* worms used in this study were N2 wild-type strain (kindly received from Prof. Limor Broday, Tel-Aviv University, Israel). The worms were maintained on nematode growth medium (NGM) plates seeded with *Escherichia coli* OP50 as food source at 20 ​°C as described previously [23,101]. The preparation of synchronized worms was performed using the alkaline hypochlorite method [91,102]. The hatched worms (L1 stage larvae) were transferred to fresh agar plates and cultured at 20 ​°C for phenotype analysis [103].

### 4.2 SWCNTs suspension

1 ​mg of HiPCO SWCNTs (NanoIntegris) were suspended with 2 ​mg single-stranded DNA sequence ((GT)_15_, Integrated DNA Technologies) in 0.1 ​M NaCl via bath sonication (Elma P-30H, 80 ​Hz for 10 ​min), followed by two cycles of direct tip sonication (QSonica Q125, 3 ​mm tip, 4 ​W for 20 ​min) in an ice bath. The resulting suspension was centrifuged for 90 ​min at 16,100 rcf twice in order to separate the individually suspended SWCNTs from aggregates and impurities. After each centrifugation step, 80% of the supernatant was collected and the pellet discarded. The absorption spectra of the suspension were recorded using an ultraviolet–visible–NIR (UV–Vis–NIR) spectrophotometer (Shimadzu UV-3600 PLUS), where sharp distinguishable peaks indicated a successful suspension (Figure S6). The concentration of (GT)_15_-SWCNT was determined spectroscopically with an extinction coefficient [104] of *ε*_632 nm_ ​= ​0.036 ​L ​mg ^−1^ · cm ^−1^.

### 4.3 Fluorescence imaging

For live fluorescence imaging, worms were mounted with 0.2 ​mM levamisole on a 3% agarose gel pad on a glass slide, covered with a coverslip, and sealed with wax. Images were taken via an inverted fluorescence microscope (Olympus IX83) at two different magnifications: 20 ​× ​, 0.7 NA (Plan FL), and 100 ​× ​, 1.3 NA (Plan FL). Visible autofluorescence was excited with an LED illumination system (CoolLED, pE4000) choosing 4 different channels covering the visible wavelength range (365 ​nm; 460 ​nm; 525 ​nm; 635 ​nm). A super-continuum white-light laser (NKT-photonics, Super-K Extreme) coupled to a tunable band-pass filter (NKT-photonics, Super-K varia) was used for a wide-range excitation (400–850 ​nm). Autofluorescence was imaged using four different filter cubes, covering the visible wavelength range: DAPI (Chroma, 49000-ET-DAPI), GFP (Chroma, 49002-ET-EGFP (FITC/Cy2)), mCherry (Chroma, 49008-ET-mCherry, Texas Red), Cy5 (Chroma, 49009-ET-Cy5). Fluorescence in the visible wavelength range was detected with an EMCCD camera (Andor, iXon Ultra 888). The SWCNT-fluorescence was excited by a 730 ​nm CW laser (MDL-MD-730-1.5 ​W, Changchun New Industries). The laser excitation light was directed to the sample with a dichroic mirror (900 ​nm long-pass, Chroma) and the NIR emission of the SWCNTs was detected after an additional 900 ​nm long-pass emission filter (Chroma, ET900lp) with an InGaAs-camera (Raptor, Ninox 640 VIS-NIR). All the figures shown are representative images taken from a pool of at least 5 worm images or movies.

### 4.4 Image processing

All images were processed by ImageJ, GIMP and MATLAB. The EMCCD camera and the InGaAs camera have different pixel sizes and chip sizes. The overlay of the images from the two cameras was done by adapting the pixel sizes and the orientation, where overlay parameters of the two images were determined via a maximization of the 2D autocorrelation of an identical frame taken with both cameras. The images were then cropped to the desired size.

The analysis of number of pixels whose intensity exceeds a specific threshold (Figure S5) was done in MATLAB. The center of mass analysis was done using ImageJ.

### 4.5 Heating effect

A thermometer was placed at the focus of the objective in the same location as the worm samples were placed on top of the microscope stage. Under 730 ​nm CW laser excitation at 245 ​mW intensity, the temperature was monitored after 1, 5, and 60 ​min of laser irradiation.

### 4.6 Viability of worms exposed to SWCNTs

The worms were incubated in vials containing 100 ​μL of SWCNT in increasing concentrations: 0.1, 0.25, 0.5, 1, 5, 10, 25, 50, 100 and 300 ​mg ​L^−1^ final concentration. The highest concentration used is the concentration of the SWCNT suspension directly after suspension with (GT)_15_. OP50 was added to the vials as food source. The vials were incubated while shaking at 20 ​°C for 4 and 24 ​h. After the incubation, the worms were washed twice with 0.1 ​M NaCl to remove excess SWCNT and placed on glass slides with 0.2 ​mM levamisole on a 3% agarose closed with a cover slip and sealed with wax. Images were taken with the inverted microscope described above with the 20× objective. The worms were imaged in brightfield and the movies were processed by ImageJ (Supplementary Movie S1 and S2).

### 4.7 Optimal SWCNT concentration for imaging

Mixed population of N2 worms was washed with M9 media and transferred into vials containing 100 ​μl of 0.1 ​M NaCl or 0.5, 5, 25, 100 ​mg ​L^−1^ SWCNT diluted in 0.1 ​m NaCl, and OP50 as food source. The vials were incubated at 20 ​°C while shaking for 4 ​h. After the incubation, the worms were washed with 0.1 ​M NaCl and placed on glass slides with 0.2 ​mM levamisole on a 3% agarose gel closed with a cover slip and sealed with wax. Images were taken with the inverted microscope described above. The worms were imaged in the visible range using the CoolLED (CL-pE4000-L-SYS-20) for GFP (λ_ex_ 460, λ_em_ 525) and DAPI (λ_ex_ 365, λ_em_ 460) while NIR fluorescence was excited with the CW laser.

### 4.8 Biocompatibility assay

NGM plates were spread with 500 ​μL of SWCNTs (0.5 or 5 ​mg ​L^−1^ final concentration) or 0.1 ​M NaCl as a control. Ten Adult N2 worms, after synchronization, were picked and placed onto the spread plates and allowed to lay eggs at 20 ​°C. The number of eggs was counted after 4 ​h using a microscope (SMZ800 ​N Olympus) and movies were taken using the inverted microscope with a 20× objective (described before) in brightfield mode, after 4 ​h and 24 ​h. Movies of the plates with 5 ​mg ​L^−1^ were taken after 4 days with the 20× objective. All movies were processed by ImageJ. Four replicates were performed for each condition.

### 4.9 Fluorescence spectra

To characterize the worms’ autofluorescence in the visible range, we imaged the worms after washing with M9 media, using a fluorescence plate reader (Fusion Optics Reader Platform SPARK). The excitation and emission wavelength were λ_ex_ ​= ​365 ​nm; λ_em_ ​= ​405–900 ​nm, λ_ex_ ​= ​460 ​nm; λ_em_ ​= ​500–900 ​nm, λ_ex_ ​= ​525 ​nm; λ_em_ ​= ​565–900 ​nm and λ_ex_ ​= ​635 ​nm; λ_em_ ​= ​675–900 ​nm.

For SWCNTs characterization, we measured the fluorescence excitation emission map. Samples of 0.5 ​mg ​L^−1^ of the suspended (GT)_15_-SWNCTs, and samples of N2 worms incubated for 4 ​h with 0.1 ​M NaCl or with SWCNTs, were added to the wells of a 96 well plate. The fluorescence spectra were acquired using an inverted fluorescence microscope (Olympus IX73) coupled to a spectrograph and a liquid-nitrogen cooled InGaAs detector (HRS-300SS, and PyLoN-IR 1024-1.7, Princeton Instruments, Teledyne Technologies). A super-continuum white-light laser (NKT-photonics, Super-K Extreme) coupled to a tunable band-pass filter (NKT-photonics, Super-K varia) was used as excitation light source.

## Author contributions

Adi Hendler-Neumark: Investigation, Resources, writing original draft, Visualization. Verena Wulf: Formal analysis, Software, Writing – review & editing. Gili Bisker: Conceptualization, Writing – review & editing, Supervision, Funding acquisition.

## Declaration of competing interest

The authors declare that they have no known competing financial interests or personal relationships that could have appeared to influence the work reported in this paper.
