# Supplementary material for: In vivo imaging of fluorescent single-walled carbon nanotubes within C. elegans nematodes in the near-infrared window
Source: Mater Today Bio. 2021 Dec 2;12:100175. doi: 10.1016/j.mtbio.2021.100175 (PMC8649898; doi:10.1016/j.mtbio.2021.100175)
Supplement: Multimedia component 12 [file mmc12.pdf]

# *In vivo* imaging of fluorescent single-walled carbon nanotubes within C. elegans nematodes in the near-infrared window

Adi Hendler-Neumark[a], Verena Wulf[a] and Gili Bisker[a,b,c,d,*]

[a] Department of Biomedical Engineering, Faculty of Engineering, Tel Aviv University, Tel Aviv 6997801, Israel

[b] Center for Physics and Chemistry of Living Systems, Tel-Aviv University, Tel Aviv 6997801, Israel

[c] Center for Nanoscience and Nanotechnology, Tel-Aviv University, Tel Aviv 6997801, Israel

[d] Center for Light Matter Interaction, Tel-Aviv University, Tel Aviv 6997801, Israel

*E-mail: bisker@tauex.tau.ac.il

## Supporting Information

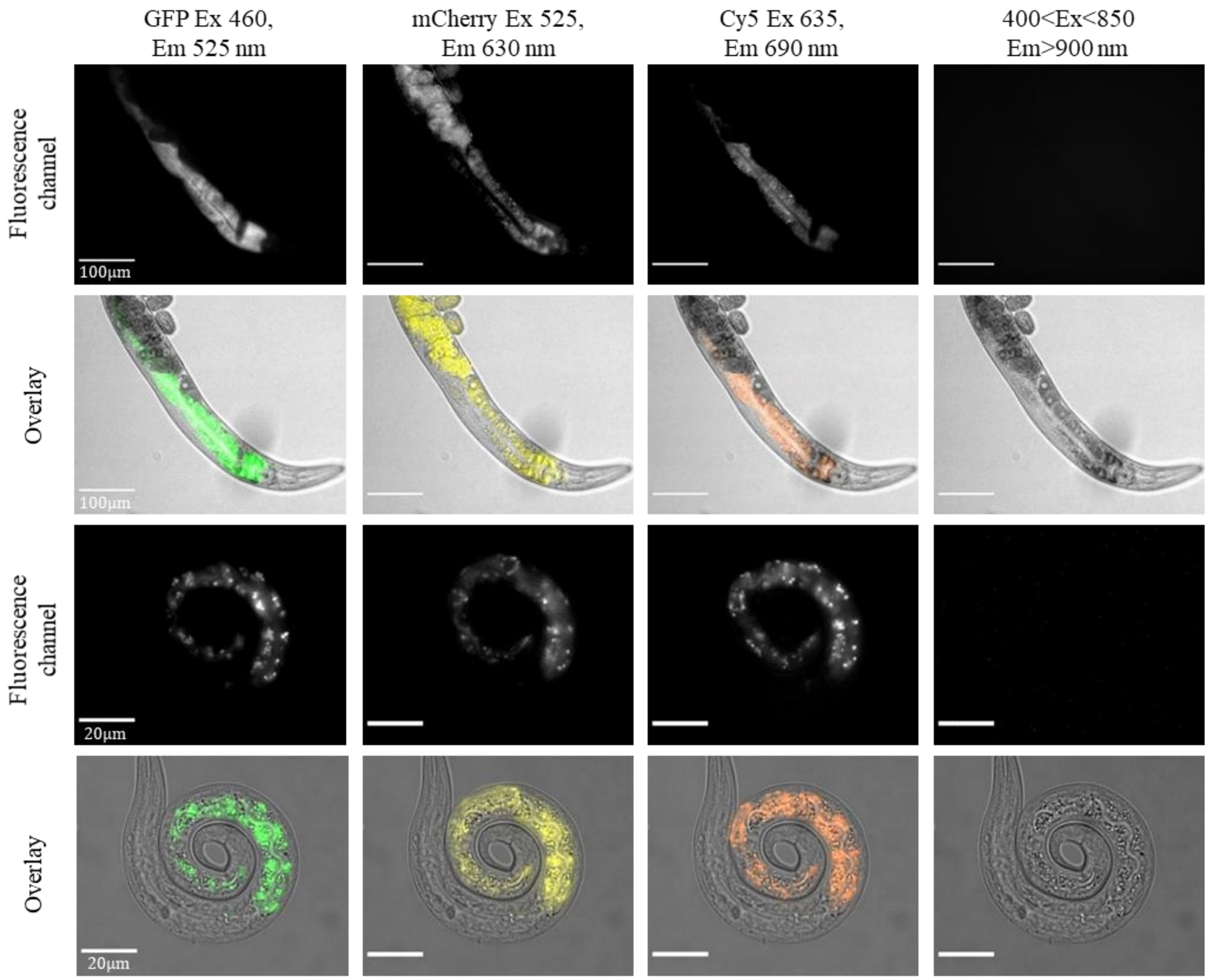


**Figure S1: Autofluorescence in the worm under excitation with a supercontinuum laser ($\lambda_{ex}$ = 400-850 nm).** Worms were imaged in the inverted imaging microscope with multiple excitation wavelengths using a supercontinuum laser. The visible autofluorescence for three fluorescence channels (GFP, mCherry, Cy5, excitation-emission filters are specified in the methods section) were acquired by an EMCCD camera, sensitive in the visible wavelength range (columns 1-3). The NIR autofluorescence (>900 nm) was imaged with an InGaAs-camera under excitation with the entire visible wavelength range (400-850 nm) (right column). The respective excitation power and exposure time for the different excitation wavelength are summed up in **Table S1** for comparison. Fluorescence emission images are shown in the top and third rows for a 20X or 100X magnification, respectively. The overlaid images of the fluorescence with the brightfield (BF) are shown in the second and bottom rows for the 20X or 100X magnification, respectively.

**Table S1: Excitation power and exposure time for images taken in Figure 1, 3, 4, S1, S2 and S3.** Red shaded rows indicate conditions for measurements of NIR fluorescence.

| **Excitation wavelength $\lambda_{ex}$** | **Excitation power (20X)** | **Exposure time $t_{ex}$ (20X)** | **Excitation power (100X)** | **Exposure time $t_{ex}$ (100X)** |
|---|---|---|---|---|
| Supercontinuum laser 460 nm ± 10 nm | 2.2 mW | 0.2 s | 3.5 mW | 0.2 s |
| Supercontinuum laser 525 nm ± 10 nm | 5.4 mW | 1.0 s | 6.8 mW | 1.0 s |
| Supercontinuum laser 635 nm ± 10 nm | 18.2 mW | 0.3 s | 23.7 mW | 10 s |
| Supercontinuum laser 400 nm - 850nm | 45.6 mW | 0.3 s | 55.6 mW | 10 s |
| LED: 365 nm | 5.5 mW | 0.01 s | 0.23 mW | 0.01 s |
| LED: 460 nm | 66.5 mW | 0.02 s | 6.5 mW | 0.02 s |
| LED: 525 nm | 4.1 mW | 0.1 s | 0.2 mW | 0.1 s |
| LED: 635 nm | 20.6 mW | 2.0 s | 2 mW | 2.0 s |
| LED: 365; 460; 525; 635 nm | 125 mW | 2.0 s | 12 mW | 2.0 s |
| CW-laser 730 nm | 181 mW | 1.0 s | 189 mW | 1.0 s |

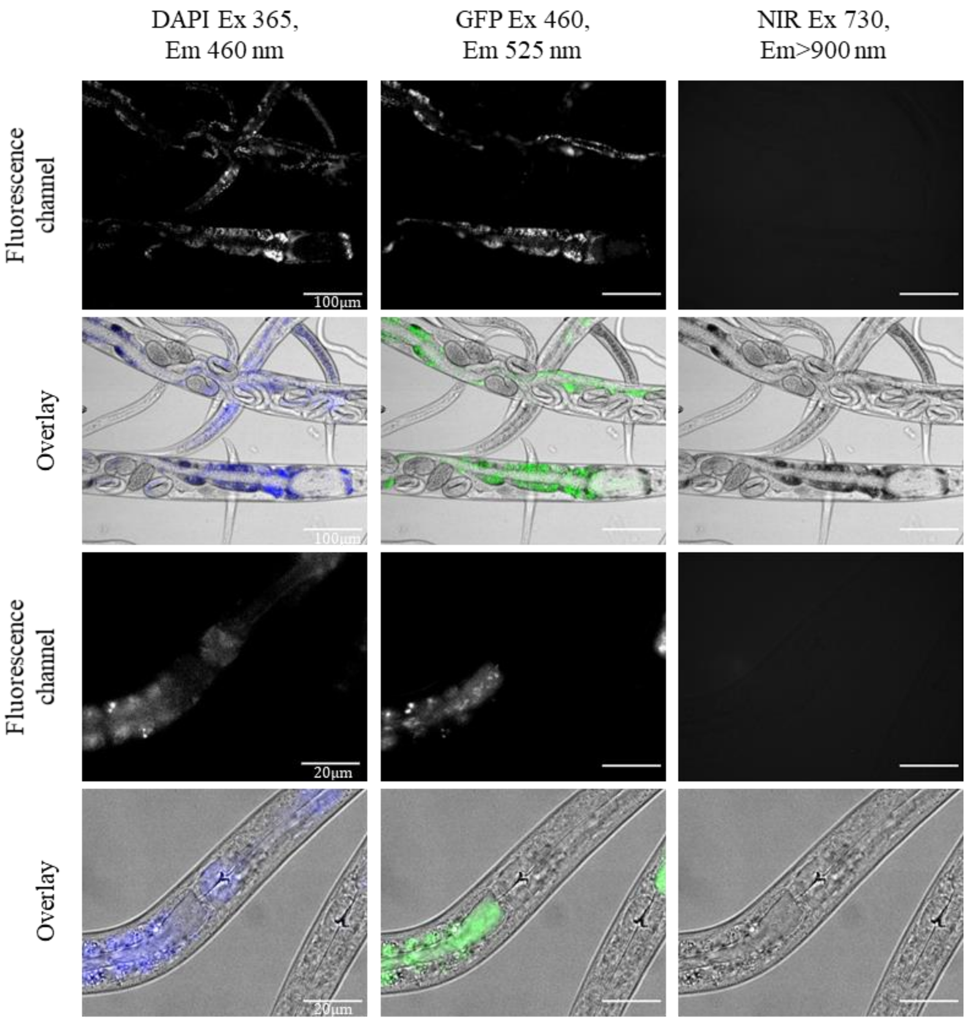


**Figure S2: Autofluorescence in the worms under LED and CW laser ($\lambda_{ex}$ = 730 nm) excitation.** Autofluorescence in the visible range of the worms' intestine granule organelles was imaged with an EMCCD-camera under excitation with an LED illumination system. The fluorescence channels are: DAPI (blue, $\lambda_{ex}$ = 365 nm; $\lambda_{em}$ = 435-485 nm; left column) and GFP (green, $\lambda_{ex}$ = 460 nm; $\lambda_{em}$ = 500-550 nm; middle column). The right column shows images taken with an InGaAs camera, detecting fluorescence in the NIR spectral region (>900 nm) under $\lambda_{ex}$ = 730 nm CW-laser excitation, demonstrating the lack of autofluorescence in the NIR. The respective excitation power and exposure time for the different excitation wavelengths are summarized in **Table S1** for comparison. Rows 1 and 3 show the fluorescence emission images, rows 2 and 4 show their overlay with the brightfield images. The scale bar in the top two rows is 100 µm and in the bottom two rows is 20 µm.

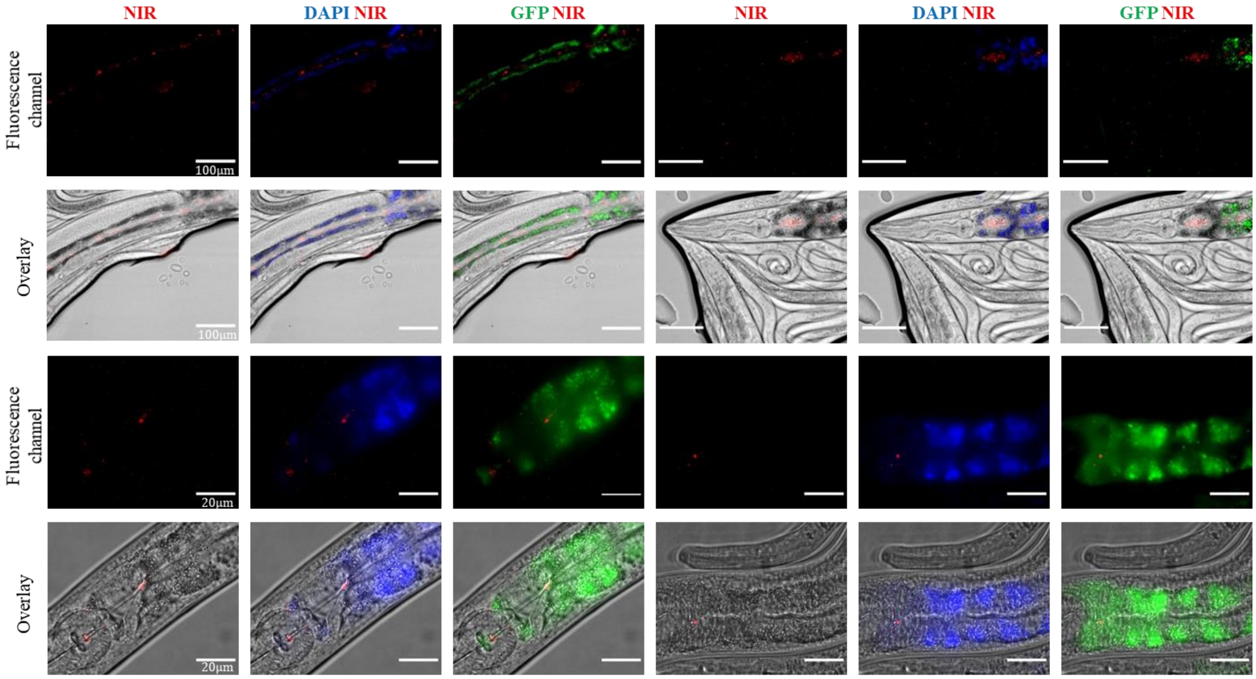


**Figure S3: SWCNT imaged inside the worms.** Fluorescence images in the NIR (red), NIR with DAPI (red and blue) or NIR with GFP (red and green) in the first and third rows. Overlaid images of the brightfield and fluorescence channels are in the second and forth row. The scale bar is 100 μm and 20 μm with 20X and 100X magnification, for the top two rows and bottom two rows, respectively.

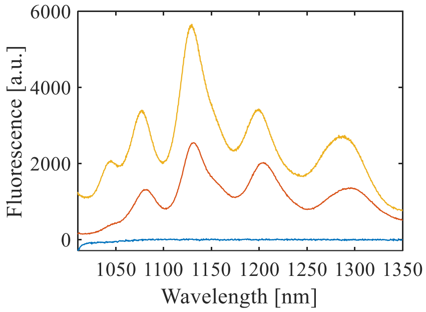


**Figure S4: Fluorescence spectra before and after digestion.** NIR fluorescence spectra under 730 nm CW laser excitation comparing the fluorescence emission of N2 worms (blue), $(GT)_{15}$-SWCNT within the N2 worms (red) and $(GT)_{15}$-SWCNT (yellow).

**SWCNT dynamic movement analysis**

The NIR movies of the SWCNT within the worms, taken with the InGaAs camera, were binarized according to a threshold intensity set according to the first frame. Subsequently, the number of white pixels in each frame was quantified, and the center of mass in each frame of the movies was calculated by ImageJ.

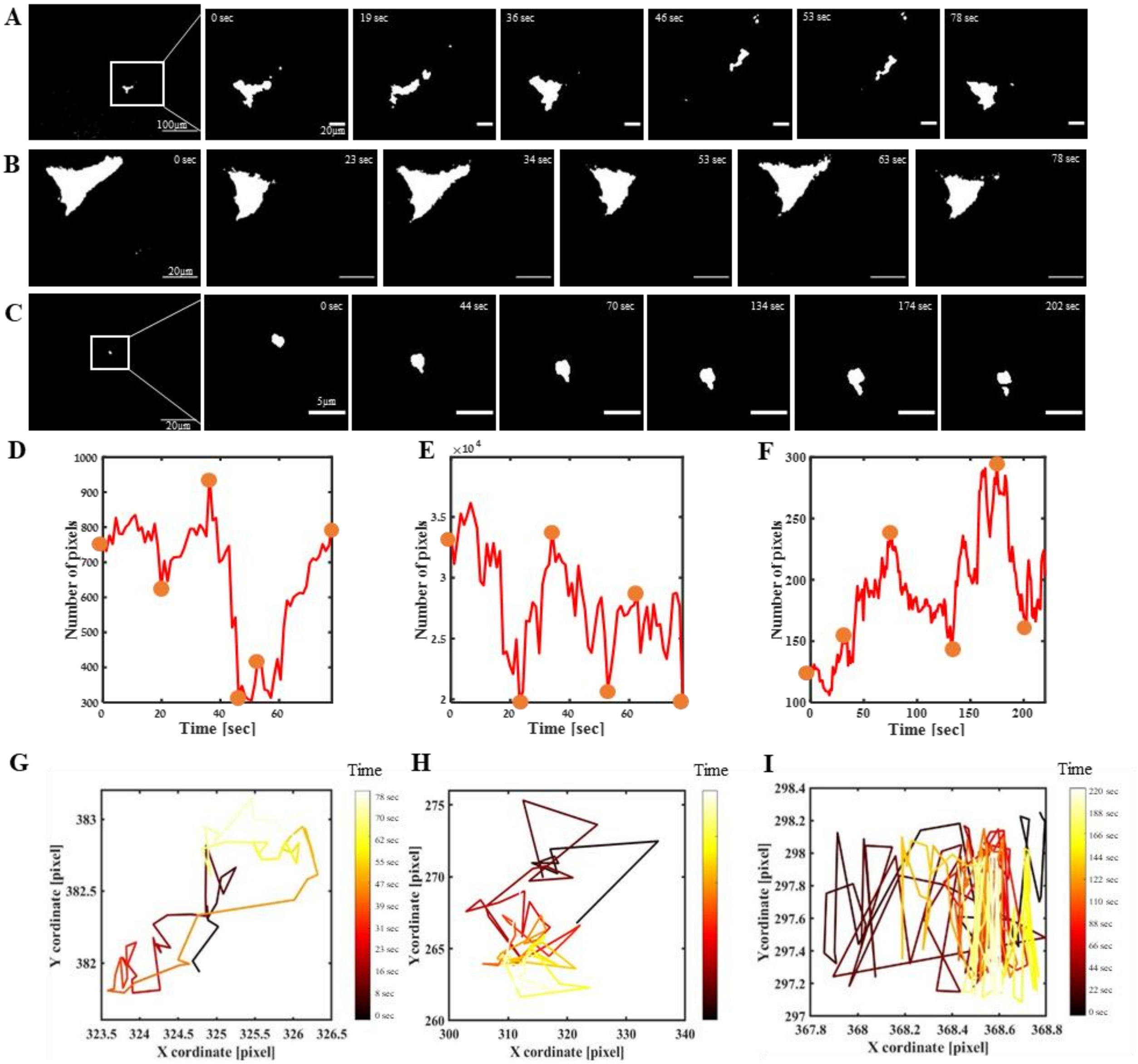


**Figure S5: SWCNT dynamics in the worms. A)-C)** Images taken from the analysis of the SWCNT fluorescence signal in the NIR channel of supplementary **Movies S9-11**. **A)** Images taken with a 20X objective, the scale bar is 100 µm in the first image. The other images in the row show the ROI marked in the first image with a scale bar of 20 µm. **B)** Images taken with a 100X

objective, the scale bar is 20 µm. **C**) Images taken with a 100X objective, the scale bar is 20 µm in the first image. The other images in the row show the ROI marked in the first image with a scale bar of 5 µm. **D**)-**F**) Total number of white pixels in each time frame of the supplementary **Movies S9-11**. The orange dots correspond to the time points shown in the images in **A**-**C**, respectively. **G**)-**I**) The center of mass coordinate of the region shown in **A**, **B** and **C**, respectively.

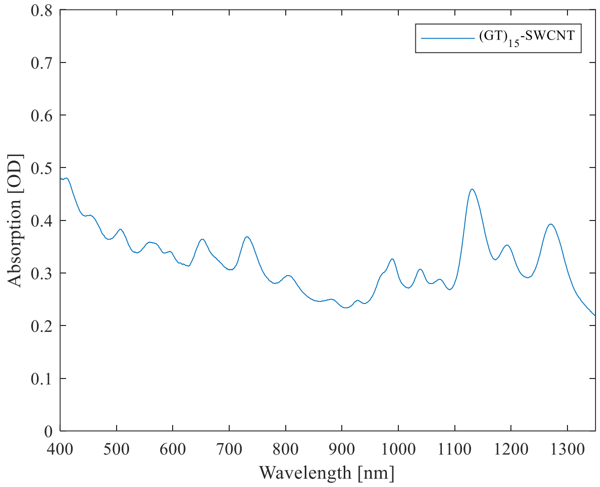


**Figure S6: $(GT)_{15}$-SWCNT absorption spectrum.**

**List of Movies**

**Movie S1:** Time-lapse video of the N2 worms following 24 hours incubation in 300 mg $L^{-1}$ $(GT)_{15}$-SWCNT. The movie was taken with a 20X magnification. Scale bar is 100 µm.

**Movie S2:** Time-lapse video of the N2 worms following 24 hours incubation in 0.1M NaCl as control. The movie was taken with a 20X magnification. Scale bar is 100 µm.

**Movie S3:** Time-lapse video of the N2 worms following 4 hours of exposure to 0.5 mg $L^{-1}$ $(GT)_{15}$-SWCNT. The movie was taken with a 20X magnification. Scale bar is 100 µm.

**Movie S4:** Time-lapse video of the N2 worms following 4 hours of exposure to 0.1M NaCl as control. The movie was taken with a 20X magnification. Scale bar is 100 µm.

**Movie S5:** Time-lapse video of the N2 worms following 24 hours of exposure to 0.5 mg $L^{-1}$ $(GT)_{15}$-SWCNT. The movie was taken with a 20X magnification. Scale bar is 100 µm.

**Movie S6:** Time-lapse video of the N2 worms following 24 hours of exposure to 0.1M NaCl as control. The movie was taken with a 20X magnification. Scale bar is 100 µm.

**Movie S7:** Time-lapse video of the N2 worms following 4 days of exposure to 5 mg $L^{-1}$ $(GT)_{15}$-SWCNT. The movie was taken with a 20X magnification. Scale bar is 100 µm.

**Movie S8:** Time-lapse video of the N2 worms following 4 days of exposure to 0.1M NaCl as control. The movie was taken with a 20X magnification. Scale bar is 100 µm.

**Movie S9:** Time-lapse video showing the dynamics of the $(GT)_{15}$-SWCNT fluorescence (red) in the intestine of N2 worms after 4 hours incubation. The movie was taken with a 20X magnification. The movie is 40 times faster than real-time. Scale bar is 100 µm.

**Movie S10 and S11:** Time-lapse video showing the dynamics of the $(GT)_{15}$-SWCNT fluorescence (red) in the intestine of N2 worms after 4 hours incubation. The movie was taken with a 100X magnification. The movie is 40 times faster than real-time. Scale bar is 20 µm.
